# High expression of PDZ-binding kinase is correlated with poor prognosis and immune infiltrates in hepatocellular carcinoma

**DOI:** 10.1186/s12957-021-02479-w

**Published:** 2022-01-22

**Authors:** Wei Mu, Yaoli Xie, Jinhu Li, Runzhi Yan, Jingxian Zhang, Yu’e Liu, Yimin Fan

**Affiliations:** 1 Department of Neurosurgery, The First Hospital of Shanxi Medical University, Taiyuan, 030001 Shanxi China; 2 Department of Interventional Radiology, Shanxi Provincial People’s Hospital, Taiyuan, 030012 Shanxi China; 3 Department of Physiology, Shanxi Medical University, Taiyuan, 030001 Shanxi China

**Keywords:** PDZ-binding kinase (PBK), Hepatocellular carcinoma (HCC), Tumor-infiltrating immune cells (TIICs), Bioinformatics analysis, Signaling pathways

## Abstract

**Background:**

PDZ-binding kinase (PBK) encodes a serine/threonine protein kinase related to the dual specific mitogen-activated protein kinase kinase (MAPKK) family. There is evidence that overexpression of this gene is associated with tumorigenesis. However, the role of PBK in hepatocellular carcinoma (HCC) remains unclear. Therefore, we evaluated the prognostic role of PBK and its correlation with immune infiltrates in hepatocellular carcinoma.

**Methods:**

The expression of PBK in pan-cancers was studied by Onconmine and TIMER. The expression of PBK in HCC patients and its relationship with clinicopathological characteristics were analyzed using The Gene Expression Profiling Interactive Analysis (GEPIA), The human protein atlas database (HPA), The Cancer Genome Atlas (TCGA), and Gene Expression Omnibus (GEO) databases. Receiver operating characteristic (ROC) curve was used to determine the diagnostic value of PBK in HCC patients. The relationship between PBK and prognosis of HCC was performed by GEPIA and Kaplan Meier plotter web tool. The correlations between the clinical characteristics and overall survival were analyzed by Univariate Cox regression and Multivariate Cox hazards regression to identify possible prognostic factors for HCC patients. LinkedOmics was applied to investigate co-expression associated with PBK and to analyze Gene Ontology (GO) and Kyoto Encyclopedia of Genes and Genomes (KEGG) pathways. The network map of PBK and related genes is constructed by GeneMANIA. Finally, TIMER and TISIDB were used to analyze the correlations between PBK and tumor-infiltrating immune cells.

**Results:**

Multiple database analysis shows that PBK was highly expressed in many types of tumors, including hepatocellular carcinoma, and was significantly related to tumor stage (*P*=0.0089), age (*P*=0.0131), and race (*P*=0.0024) of HCC patients. The receiver operating characteristic (ROC) curve analysis showed that PBK had high diagnostic potential to HCC in GSE76427 (AUC=0.8799), GSE121248 (AUC=0.9224), GSE62232 (AUC=0.9975), and GSE84402 (AUC=0.9541). Multivariate Cox hazards regression showed that high expression of PBK may be an independent risk factor for overall survival in HCC patients (HR = 1.566, 95% CI=1.062–2.311, *P*= 0.024). The Protein–protein interaction network showed that PBK significantly interacted with LRRC47, ARAF, LGALS9B, TTK, DLG1, and other essential genes. Furthermore, enrichment analysis showed that PBK and co-expressed genes participated in many biological processes, cell composition, molecular functions, and pathways in HCC. Finally, the immune infiltration analysis by TIMER and TISIDB indicated that a significant tightly correlation between PBK and macrophages, neutrophils, as well as chemokines and receptors.

**Conclusions:**

High expression of PBK is significantly correlated with poor survival and immune infiltrates in hepatocellular carcinoma. Our study suggests that PBK can be used as a biomarker of poor prognosis and potential immune therapy target in hepatocellular carcinoma.

**Supplementary Information:**

The online version contains supplementary material available at 10.1186/s12957-021-02479-w.

## Introduction

Liver cancer is the sixth most common cancer and the fourth leading cause of cancer-related death in the world [1]. Hepatocellular carcinoma (HCC) accounts for about 75–85% of primary liver cancer and is a major health problem worldwide [2]. The causes of liver cancer occurrence and death are Hepatitis B virus (HBV) (33%), alcohol (30%), hepatitis C virus (HCV) (21%), and other causes (16%) [1]. In the early stage of HCC (TNM stage I), treatment includes liver resection, liver transplantation, and ablation, and the prognosis is satisfactory. In the medium term (TNM stage II or III), palliative locoregional treatment such as transcatheter arterial chemoembolization (TACE) remains the gold standard. However, most cases of HCC are diagnosed in the late stage (TNM stage III or IV). At that time, surgical treatment or TACE is no longer available and systematic treatment may be a better choice, but the prognosis is still poo r[3].

With the development of DNA sequencing technology, precision medicine has made great progress, and many new therapeutic targets have been recognized. PDZ-binding kinase (PBK) is a member of the mitogen-activated protein kinase (MAPK) family, which acts on oncogenes as upstream kinases in some important signaling pathways [4]. PBK is highly expressed in testicular and fetal samples, but rarely found in other normal tissues [5]. Physiologically, through phosphorylation of various targets, it plays an active regulatory role in the proper separation of chromosomes and cytokinesis [6]. Current studies have shown that the increased expression of PBK is closely related to the occurrence and development of multiple tumors [7, 8], including lung cancer [9], ovarian carcinoma [10], oral cancer [11], prostate cancer [12], and gastric carcinoma [13]. Therefore, the relationship between the expression of PBK and the prognosis of HCC, as well as the correlation between PBK and immune regulation is worth exploring.

In this study, we revealed the expression of PBK in pan-cancers and HCC. The results showed that PBK expression was extremely higher in most common tumor tissues, including liver cancer, from Oncomine and TIMER databases (Fig.S1 A&B). The mRNA and protein expression patterns of PBK were noticeably upregulated in HCC compared to normal tissue (Fig.S1 C-F). Then these results were verified by the GEO database as well. In addition, bioinformatics analysis showed that high expression of PBK was associated with poor prognosis in HCC. Subsequently, we explored the association of PBK expression and immune infiltrating via TIMER and TISIDB databases. These findings will help to understand the potential role of PBK in tumor immunology and its prognostic value.

## Materials and methods

### Data acquisition

The RNA-seq data of HCC from The Cancer Genome Atlas (TCGA) was downloaded from the cBioPortal platform [14, 15]. Clinicopathological information, including gender, age, tumor grade, race, and survival data were also retrieved from the platform; patients who lacked complete information were excluded. We also downloaded the microarray dataset GSE84402, GSE76427, GSE62232, and GSE121248 from the Gene Expression Omnibus (GEO) database. In total, data relating to HCC in the TCGA related to 311 specimens; GSE84402 featured 14 samples of HCC tissue and 14 adjacent non-tumor specimens; GSE76427 featured 115 samples of HCC tissue and 52 adjacent non-tumor specimens; GSE62232 featured 81 samples of HCC tissue and 10 adjacent non-tumor specimens; GSE121248 featured 70 samples of HCC tissue and 37 adjacent non-tumor specimens. Both mRNA expression profiles and clinical information are open-access and publicly available. Therefore, this study did not need approval from the Ethics Committee.

### The expression of PBK in pan-cancers

To investigate the expression of PBK gene in pan-cancers, two online databases were used respectively—Oncomine and TIMER. There are 715 datasets and 86733 specimens collected by Oncomine web applications (https://www.oncomine.org/) [16]. These datasets come from public repositories, such as GEO, TCGA dataset, etc. In this study, we used Oncomine to analyze the expression of PBK in pan-cancers with *P* value:0.05, the fold change: 2, gene rank: top10%, data type: DNA+mRNA. TIMER (http://timer.comp-genomics.org/) [17, 18] is a comprehensive resource for systematical analysis of immune infiltrates across diverse cancer types, which allows us to study the differential expression between tumor and adjacent normal tissues for PBK across all TCGA tumors by Gene_DE module. The statistical significance computed by the Wilcoxon test is annotated by the number of stars (*: *p* value < 0.05; **: *p* value <0.01; ***: *p* value <0.001).

### Expression profile and diagnostic value of PBK in HCC and normal tissue

HCCDB (http://lifeome.net/database/hccdb/) [19] is a database of hepatocellular carcinoma expression atlas, which consists of 15 public HCC expression datasets with up to about 4000 clinical samples. Gene Expression Profiling Interactive Analysis (GEPIA) (http://gepia.cancer-pku.cn/) [20] is a newly developed interactive web server for analyzing the RNA sequencing expression data of 9736 tumors and 8587 normal samples from the TCGA and the GTEx projects, which provides interactive functions, including differential expression analysis, mapping, correlation analysis, and survival analysis, etc. In this study, we used these online tools to analyze the expression of PBK in HCC and normal tissue. The human protein atlas database (HPA) (https://www.proteinatlas.org/ )[21] provides tissue and cell distribution information of all 24,000 kinds of human proteins and provides free public query. We used this online database to explore the protein expression of PBK in HCC and normal tissues. We also downloaded PBK expression profiles from 4 independent GEO databases to validate the expression of PBK and to assess the diagnostic value of PBK by receiver operating characteristic (ROC) curves and the area under the curve (AUC) in HCC. Finally*,* the correlation analysis of PBK and clinicopathological features according to the TCGA-LIHC dataset were examined.

### Prognostic analysis from online database and TCGA-LIHC clinical information

GEPIA can provide an association between PBK gene expression level and prognosis in patients with LIHC. The analysis indicators of GEPIA included Overall Survival and Disease-Free Survival, and the group cut-off is median. Kaplan Meier plotter (http://kmplot.com/analysis) [22] is a friendly website for online analysis of the impact of genes on the survival of cancer patients. It included 364 HCC patients for overall survival (OS) analysis, 316 for recurrence-free survival (RFS) analysis, 370 for progression-free survival (PFS) analysis, and 362 for disease-specific survival (DSS) analysis. In addition, A total of 311 HCC patients was included in the overall survival (OS) analysis from the TCGA-LIHC database. Median expression of PBK of all samples was chosen as a cut-off to divide samples into the PBK-high (*n* = 155) group and PBK-low (*n* = 156) group. The Kaplan-Meier analysis was performed to investigate the prognosis of patients in the two groups. Univariate Cox regression and Multivariate Cox hazards regression analysis were performed to identify possible prognostic factors for HCC patients.

### GeneMANIA

GeneMANIA (http://genemania.org/ )[23] is an online site for discovering relevant genes and building visual networks of protein-protein interactions (PPI). The network map of PBK and related genes is constructed by GeneMANIA, which can also show the mode of interaction and functional analysis. In this map, each small circles represent different proteins in the network, the size of the circle represents the strength of the interaction, different colors of the attachment have different means of interaction.

### Linkedomics

LinkedOmics (http://www.linkedomics.org/ )[24] is an open and free online data analysis website, which contains multi omics data of all 32 TCGA cancer type s[24]. We used the LinkFinder module of LinkeDomics website to find genes closely related to PBK in TCGA cohort, and displayed in the form of a volcano and heat map. Besides, LinkInterpreter module of LinkedOmics was applied to conducted analyses of Gene Ontology (Biological Process, Cell Composition, Molecular Function) and Kyoto Encyclopedia of Genes and Genomes (KEGG) pathways through Gene Set Enrichment Analysis (GSEA).

### Tumor Immune Estimation Resource (TIMER) Database

The version of TIMER2.0 provides immune infiltrates' abundances estimated by multiple immune deconvolution methods, and allows users to generate high-quality figures dynamically to explore tumor immunological, clinical and genomic features comprehensively [25]. Online analysis gene module was used to investigate the correlation between PBK expression and the abundance of CD4+T cells, CD8+T cells, B cells, macrophages, neutrophils, and dendritic cells by correcting part of Spearman correlation with tumor purity. Then, we used the outcome module to explore Kaplan-Meier curves for the corresponding immune infiltrates and HCC. Prognostic factors were assessed using a multivariable Cox proportional hazard model.

### Tumor-Immune System Interaction Database (TISIDB)

TISIDB (http://cis.hku.hk/TISIDB/) [26] is an online web integrated repository portal for tumor immune system interaction. In this study, we performed TISIDB to determine the correlations between the expression of PBK and chemokines and receptors across human cancers.

### Statistical analysis

All statistical analyses and graphs were performed in SPSS21.0, GraphPad Prism 8 software, and web resources. The measurement data are presented as mean ± SD. Unpaired *T* tests were used to analyze the differential expression levels of PBK mRNA between the HCC tissues and the normal tissues from the GEO databases. ROC curve was performed to detect the diagnostic value of PBK in HCC. Kaplan-Meier and log-rank tests were conducted to assess the effect of PBK on survival. Univariate and multivariate analyses were performed using Cox proportional hazards regression model. Statistically significant differences were considered when *P* < 0.05.

**Fig. 1:**
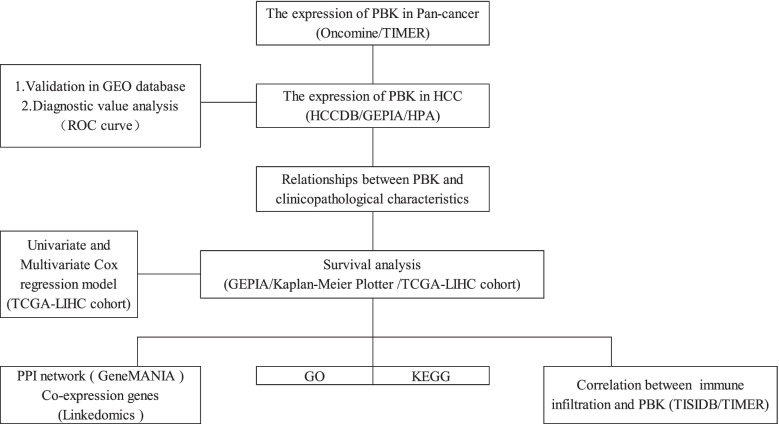
Work flow chart of this study

This study was conducted according to the flow chart shown in Fig. 1.

## Results

### Validation from the GEO database and the diagnostic value of PBK in HCC

**Fig. 2:**
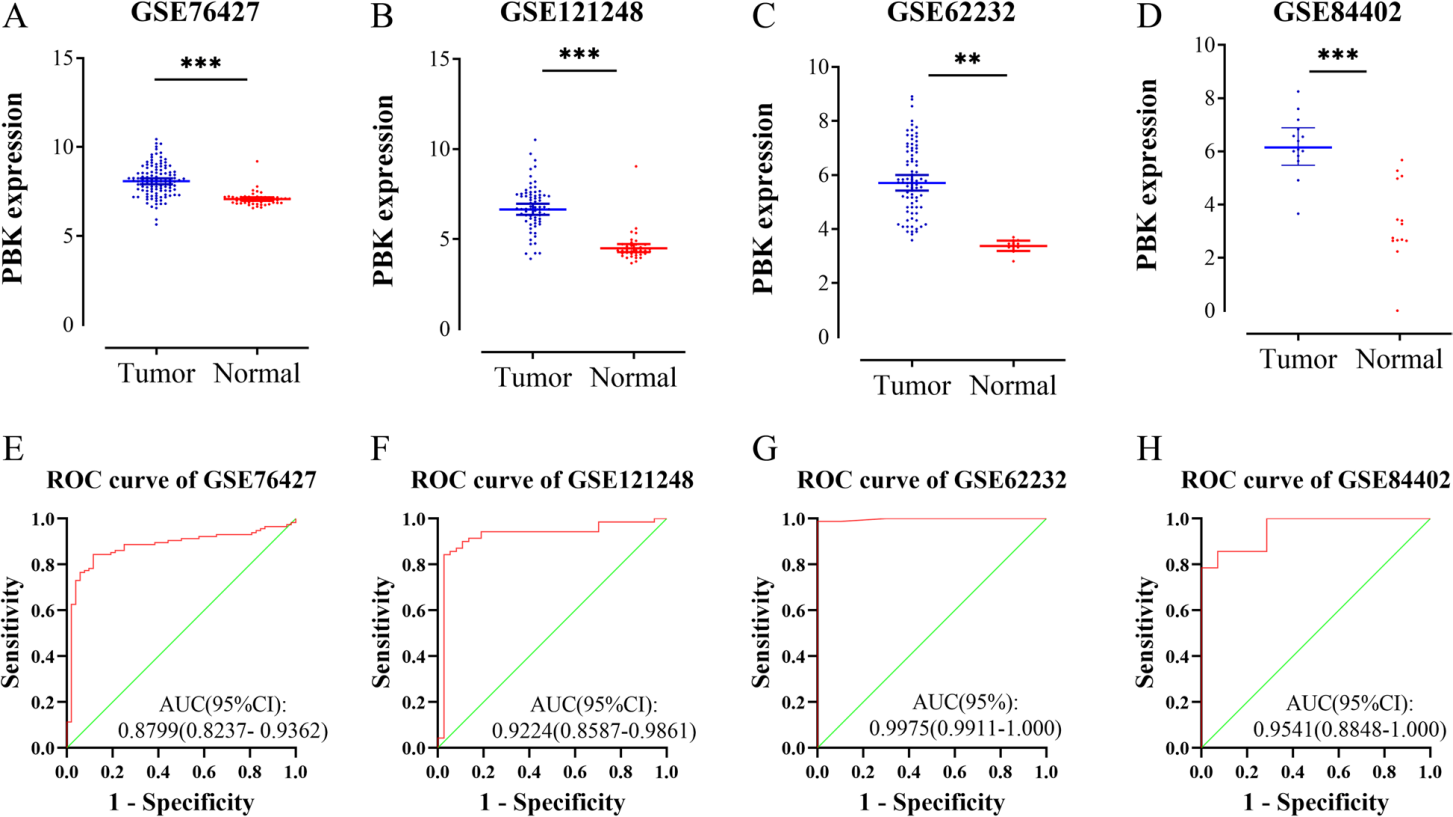
The expression and diagnostic significance of PBK in the GEO database. Levels of PBK are statistically higher in HCC than that in normal tissues in GSE76427 (**A**), GSE12148 (**B**), GSE62232 (**C**), and GSE84402 (**D**). (^***^P<0.001, ^**^P<0.01, ^*^P<0.05) ROC curves of PBK for the identification of HCC were meaningful in GSE76427 (**E**), GSE12148 (**F**), GSE62232 (**G**), and GSE84402 (**H**) (*P*<0.001)

We used 4 independent GEO databases to verify PBK mRNA expression in HCC and matched normal tissues. Compared to normal tissues, PBK mRNA levels were upregulated in HCC tissues, based on GSE76427 (Fig.2A), GSE121248 (Fig.2B), GSE62232 (Fig.2C), and GSE84402 (Fig.2D). The receiver operating characteristic (ROC) curves analyses were used to assess the diagnostic value of PBK in HCC (Fig.2E–H). The results showed that PBK mRNA had high diagnostic potential to HCC in GSE76427 (AUC=0.8799), GSE121248 (AUC=0.9224), GSE62232 (AUC=0.9975), and GSE84402 (AUC=0.9541).

### Relationships between PBK and clinicopathological characteristics of HCC patients

**Fig. 3:**
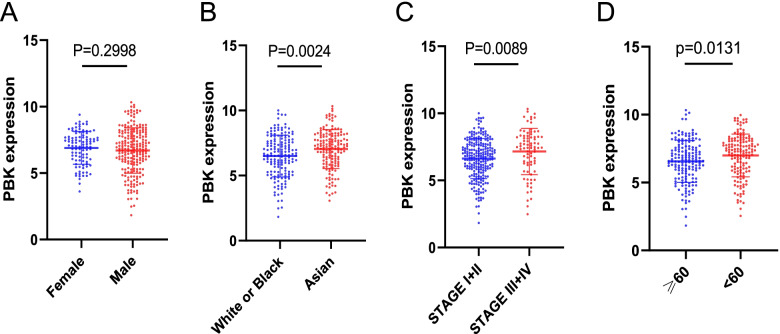
Relationships between PBK mRNA levels and clinical pathological characteristics. PBK mRNA levels was significantly correlated with race (**B**), tumor stage (**C**), and age (**D**), except for gender (**A**)

The association between PBK mRNA expression and the clinicopathological characteristics of HCC patients including gender, race, tumor stage, and age was investigated using TCGA data. Based on the median values of PBK mRNA level, 155 patients were classified as high PBK expression group, and 156 patients were classified as low PBK expression group. As shown in Fig. 3, PBK expression was significantly correlated with race (*P*=0.0024), tumor stage (*P*=0.0089), and age (*P*=0.0131), except for gender (*P*=0.2998) in HCC patients.

### Survival analysis

**Fig. 4:**
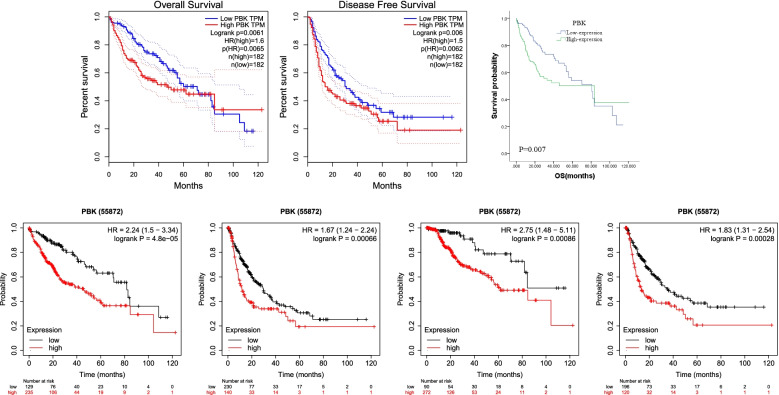
Relationship between PBK expression and poor prognosis in HCC. **A** Effect of PBK expression on HCC Overall Survival from GEPIA. **B** Effect of PBK expression on HCC disease-free survival from GEPIA. **C** Kaplan-Meier survival curves indicated that high PBK mRNA expression had a shorter OS than those with low-level of PBK from TCGA-LIHC database. **D**–**G** Effect of PBK expression on HCC survival from Kaplan-Meier Plotter (**D**: OS, **E**: DSS, **F**: RFS, **G**: PFS)

GEPIA and Kaplan-Meier Plotter were used for online survival data analysis. The results from GEPIA showed that the survival time of the group with high PBK expression was significantly shorter than that of the group with low PBK expression, both in OS (Logrank *P*=0.0061) and DFS (Logrank *P*=0.006) (Fig. 4A, B). According to the Kaplan-Meier Plotter website analysis for survival, patients having high PBK expression were associated with poor prognosis compared with those having low PBK expression (OS: Logrank *P* = 4.8e−05; DSS: Logrank *P*=0.00086; RFS: Logrank *P*=0.00028; PFS: Logrank *P*=0.00066) (Fig.4D–G).

The Kaplan-Meier analysis was performed to investigate the relationship between PBK expression and overall survival in 311 HCC patients in the TCGA cohort. Median expression of PBK of all samples was chosen as a cut-off to divide samples into the PBK-high (*n* = 155) group and PBK-low (*n* = 156) group. As shown in Fig. 4C, HCC patients with high-expression of PBK exhibited a poorer overall survival rate compared with the low-expression group (*P* = 0.007).

### Validation of PBK as an independent prognostic factor

Using the Univariate Cox regression analysis, tumor stage (HR = 2.389, 95% CI = 1.621–3.522, *P* < 0.001) and PBK expression (HR = 1.699, 95% CI = 1.154–2.502, *P* = 0.007) were identified to be related to the overall survival of 311 HCC patients in the TCGA training set. In the Multivariate Cox regression model, tumor stage (HR = 2.274, 95% CI = 1.538–3.362, *P* < 0.001) and PBK expression (HR = 1.566, 95% CI = 1.062–2.311, *P* = 0.024) were likewise confirmed to be independent prognostic factors of overall survival in HCC patients (Table 1). Overall, our results suggested that high PBK expression was an adverse prognostic factor and independent prognostic marker.

**Table 1:** Univariable and multivariable Cox regression analyses of clinical characteristics

Variable	Univariable Cox regression		Multivariable Cox regression	
	HR (95%CI)	*P* value	HR (95%CI)	*P* value
Gender	0.872 (0.717–1.061)	0.173		
Age	0.811 (0.552–1.191)	0.285		
Stage	2.389 (1.621–3.522)	<0.001	2.274 (1.538–3.362)	<0.001
Race	0.770 (0.519–1.142)	0.194		
PBK	1.699 (1.154–2.502)	0.007	1.566 (1.062–2.311)	0.024

### PBK co-expression and regulatory networks in HCC

**Fig. 5:**
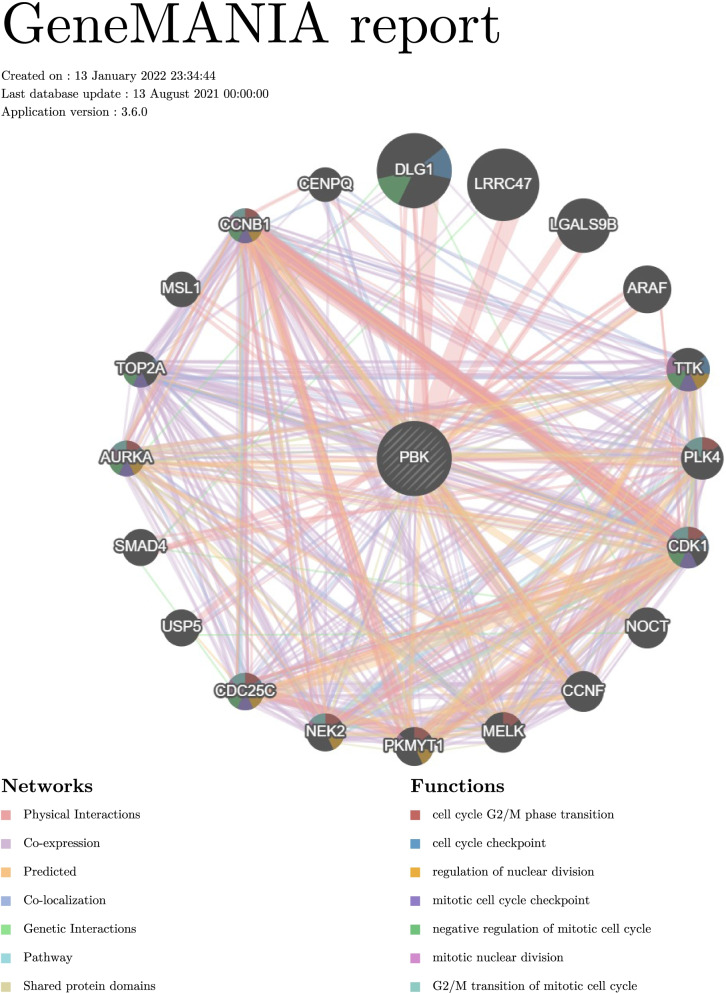
Protein-protein interaction (PPI) network and functional analysis showing the gene set enriched in the target network of PBK (GeneMANIA). Distinct colors of the network edge indicate the bioinformatics methods applied: physical interactions, co-expression, predicted, co-localization, pathway, genetic interactions, and shared protein domains. The distinct colors for the network nodes show the biological functions of the sets of enrichment genes

We used GeneMANIA to construct a protein-protein interaction (PPI) network to analyze the interaction between PBK and other genes. PPI network showed that PBK significantly interacted with LRRC47, ARAF, LGALS9B, TTK, DLG1, and other essential genes. Biological function of these genes may include cell cycle G2/M phase transition, cell cycle checkpoint, regulation of nuclear division, mitotic cell cycle checkpoint, negative regulation of mitotic cell cycle, mitotic nuclear division and G2/M transition of mitotic cell cycle (Fig. 5).

**Fig. 6:**
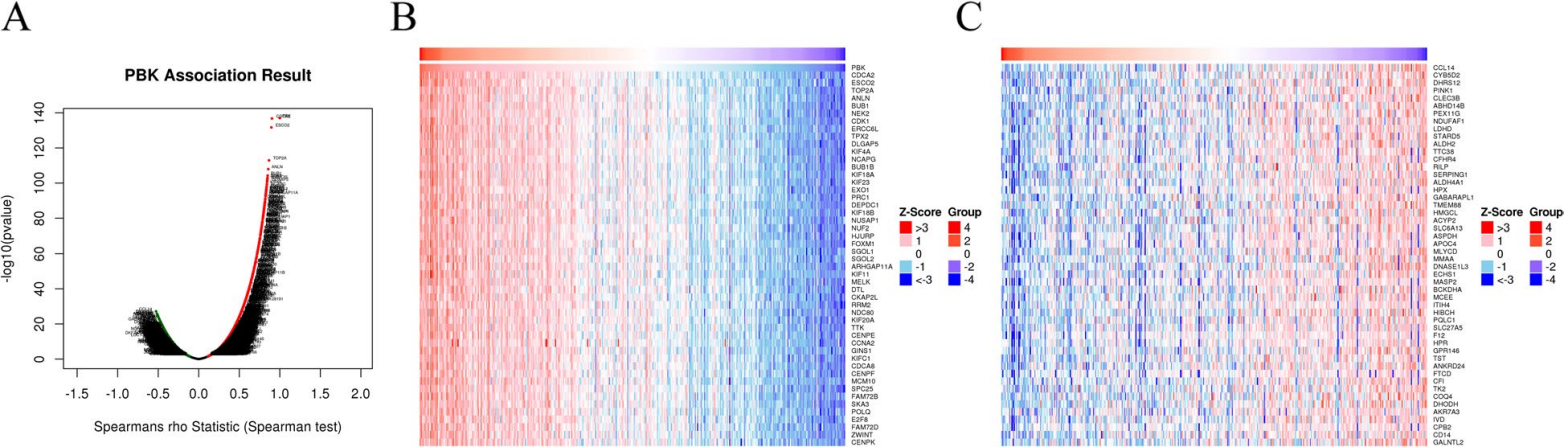
Genes differentially expressed in correlation with PBK in HCC (LinkedOmics). **A** The volcanic map showed correlations between PBK and genes differentially expressed in HCC. Red indicates positively correlated genes, and green indicates negatively correlated genes. **B**, **C** Heat maps showing genes positively and negatively correlated with PBK in HCC (TOP 50)

In addition, the co-expression genes in TCGA-LIHC that are closely related to PBK were explored on the Linkedomics website. As shown in Fig. 6A, 6869 positively correlated genes represented by red dots are closely related to PBK, while 3350 negatively correlated genes represented by green dots are closely related to PBK. The top 50 significant genes that positively and negatively correlated with PBK are shown in the heat map (Fig. 6B, C).

### Enrichment analysis of PBK functional networks in HCC

**Fig. 7:**
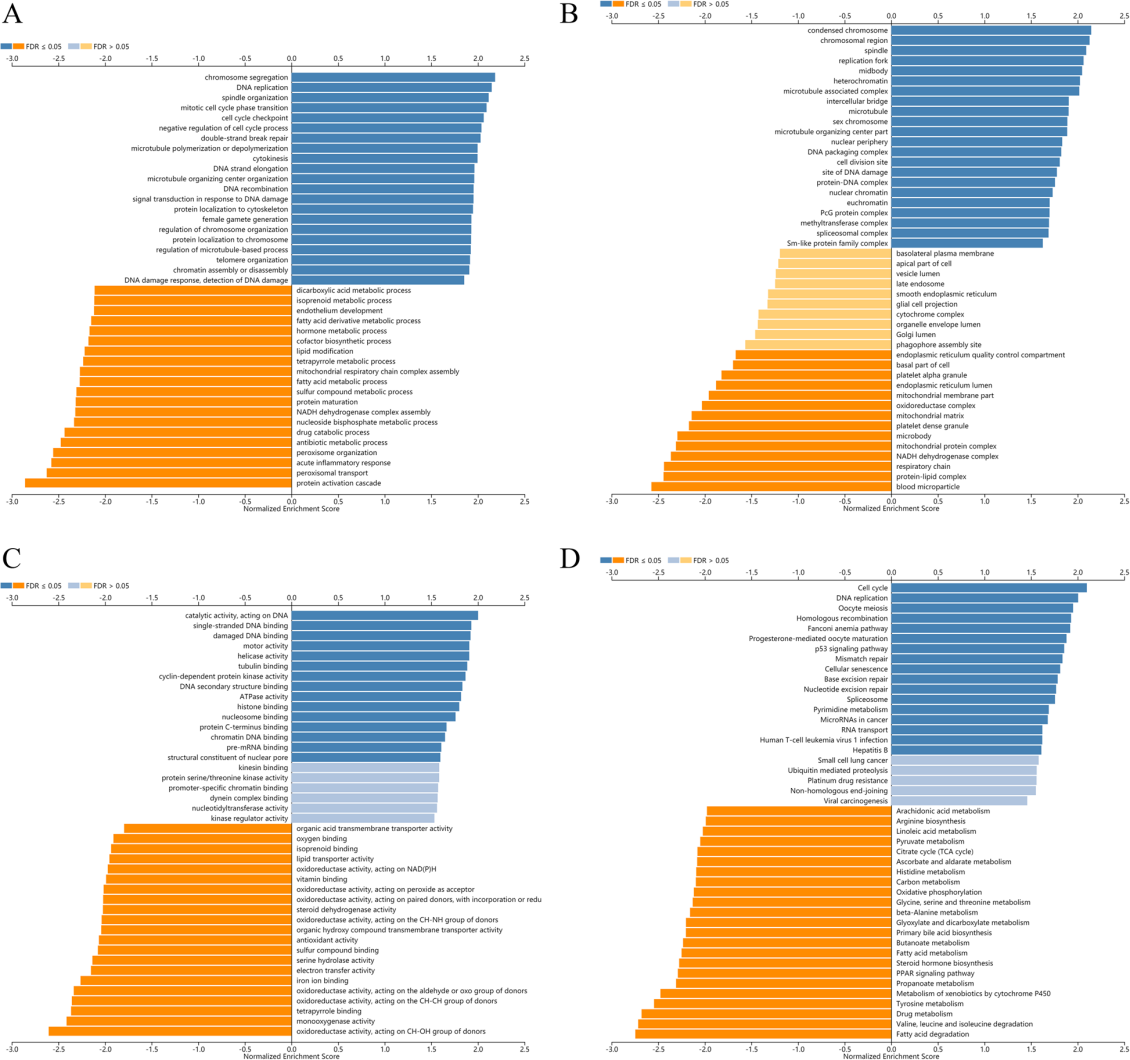
GO annotations and KEGG pathways of PBK in HCC (LinkedOmics). **A** Biological processes. **B** Cellular components. **C** Molecular functions. **D** KEGG pathway analysis

GO and KEGG analysis of PBK and co-expression genes were further analyzed using the GSEA in Linkedomics (Fig. 7). Consequently, the GO enrichment analysis revealed (1) In biological processes (BP), chromosome segregation, DNA replication, spindle organization, mitotic cell cycle phase transition, cell cycle checkpoint, protein activation cascade, peroxisomal transport, acute inflammatory response, peroxisome organization, and antibiotic metabolic process were the most significant. (2) In cell composition (CC), condensed chromosome, chromosomal region, spindle, replication fork, midbody, blood microparticle, protein-lipid complex, respiratory chain, NADH dehydrogenase complex, and mitochondrial protein complex were the most significant. 3) In molecular function (MF), catalytic activity acting on DNA, single-stranded DNA binding, damaged DNA binding, motor activity, helicase activity, oxidoreductase activity acting on CH-OH group of donors, monooxygenase activity, tetrapyrrole binding, oxidoreductase activity acting on the CH-CH group of donors and oxidoreductase activity acting on the aldehyde or oxo group of donors were the most significant. In the results of KEGG pathway analysis, PBK and co-expression genes were significantly enriched in cell cycle, DNA replication, oocyte meiosis, homologous recombination, fanconi anemia pathway, fatty acid degradation, valine, leucine and isoleucine degradation, drug metabolism, tyrosine metabolism, and metabolism of xenobiotics by cytochrome P450.

### Correlation between tumor immune cell infiltration and PBK expression in HCC

We evaluated the correlation between PBK expression in pan-caners and chemokines and receptors in the TISIDB database (Fig.S2A, Fig.S3A). As shown in Fig.S2B, PBK expression was positively closely related with CCR6 (rho=0.123, *P*=0.0175) and CCR10 (rho=0.175, *P*=0.000712), and was negatively correlated with CCR7 (rho= − 0.134, *P*=0.0098) and CXCR1(rho= − 0.188, *P*=0.000259). In addition, the result showed that 12 types of chemokines were negatively correlated with the expression of PBK, and 6 types of chemokines were positive (Fig.S3B).

**Fig. 8:**
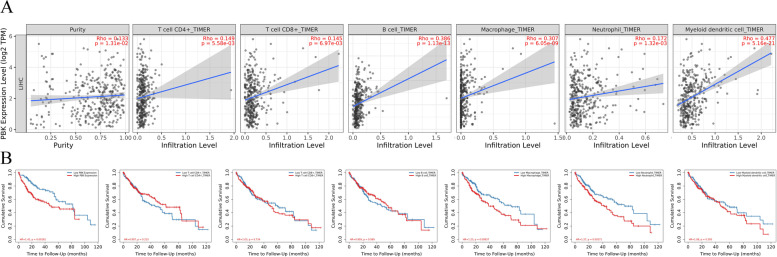
Correlation between PBK expression and TIICs and K-M plot of TIICs using data from TIMER. **A** Correlation between TIICs and PBK expression in HCC. TIICs include B cells, CD4+ T cells, CD8+ T cells, neutrophils, macrophages, and dendritic cells. **B** Kaplan-Meier survival analysis of PBK and TIICs. TIICs, tumor-infiltrating immune cells

In the TIMER database, we investigated whether PBK was correlated with six main infiltrating immune cells (CD4+T cells, CD8+T cells, B cells, macrophages, neutrophils, and dendritic cells) in HCC. The results show that a significant positive correlation between PBK and CD4+T cells (rho=0.149, *P*=5.58e−03), CD8+T cells (rho=0.145, *P*=6.97e−03), B cells (rho=0.386, *P*=1.13e−13), macrophages (rho=0.307, *P*=6.05e−09), neutrophils (rho=0.172, *P*=1.32e−03), and dendritic cells (rho=0.477, *P*=5.16e−21) (Fig. 8A). Moreover, we evaluated the relationship between PBK and six kinds of immune cells and the prognosis of HCC patients. We found that patients with high expression of PBK had a poor prognosis (*P*=0.00181), which also confirmed our previous results. Furthermore, the expression of macrophages and neutrophils was positively correlated with poor prognosis (macrophages, P=0.00837; neutrophils, P=0.00271), meanwhile, there was no correlation between the remaining immune cells (CD4+T cells, CD8+T cells, B cells, and dendritic cells) and prognosis (*P*>0.05) (Fig. 8B). Multivariate Cox regression analysis showed that macrophages (HR= 10.217, *P* value=0) and neutrophils (HR= 4.757, *P* value=0.018) were also significant independent risk factors among the six types of immune cells (Table S1).

## Discussion

Although many people, especially those with chronic liver diseases, have realized the importance of early diagnosis of liver cancer, in the countries with high incidence rates of liver cancer, such as China, Mongolia, and Egypt, most patients are diagnosed at late stages with poor prognosis. In fact, surgical or locoregional therapies can not meet the clinical needs of patients with HCC. Fortunately, sorafenib, the first targeted treatment drug, was launched in 2007, which opened the prelude of systematic treatment of HCC [27]. Systematic treatments for HCC changed in recent years with the advent of new targeted therapeutic agents and immunotherapy [28]. While these agents offer new hope for HCC patients, the optimal choice and sequence of treatment remain unknown, and without established biomarkers [29]. Therefore, it is very important to identify novel prognostic biomarkers and immune-associated therapeutic targets in HCC patients.

T-lymphokine-activated killer-cell-originated protein kinase (TOPK), also known as PDZ-binding kinase (PBK), is a novel mitotic serine/threonine protein kinase, which was confirmed to be associated with the development, progression, and metastasis of malignancies and to be a potential therapeutic target in cancer therapy [30]. Studies have shown that PBK expression is increased in various cancers compared with normal tissues [31]. In this study, we explored the expression of PBK in pan-cancers using oncomine and TIMER databases. The result has shown that PBK expression was extremely higher in most common tumor tissues, such as colon adenocarcinoma, cholangiocarcinoma, breast invasive carcinoma, liver hepatocellular carcinoma, and so on. These conclusions suggest that the possibility of PBK to be a cancer therapeutic target. Then, mRNA expression profiles and clinicopathological data of HCC were downloaded from publicly available data repositories, including TCGA and GEO. Analysis results from HCCDB, GEPIA, and HPA database showed that PBK increased significantly in HCC, both at the mRNA and protein levels. We also confirmed this with 4 independent GEO datasets- GSE84402, GSE76427, GSE62232, and GSE121248. To evaluate the prognostic value of PBK on the overall survival of HCC patients in the TCGA cohort, the Kaplan-Meier and Cox regression analyses were performed. We found out that HCC patients with high PBK expression exhibited a poorer overall survival rate compared with the low-expression group. These results indicated that PBK could act as an independent prognostic marker in HCC. Previous studies showed that PBK, as differentially expressed genes (DEGs), is highly expressed in HCC and is associated with poor prognosis, which is according to our results [32, 33]. However, our study attempted to deeper explore the relationships between PBK and clinicopathological characteristics of HCC patients. It’s the first time to reveal that the expression of PBK is closely related to age, race, and tumor stage. Patients were further stratified into subgroups of male and female, white or black and Asian, age <60 and age ≥60 years old, stage I+II and stage III+IV. Except for the subgroup of gender, the expression of PBK is higher in Asian patients under 60 with tumor stageIII+IV. The revelation of these results is helpful for the formulation of individualized treatment strategies with PBK as a therapeutic target. The receiver operator characteristic (ROC) curve and AUC estimates can determine the expression of PBK to diagnose the value of HCC. The ROC curves based on the TCGA database in AFP-negative HCC patients showed the AUC of PBK was 0.929 [34]. In the current study, the ROC curves analysis based on the GEO database shows that the AUC of PBK in GSE84402, GSE76427, GSE62232, and GSE121248 was 0.9541, 0.8799, 0.9975, and 0.9224, respectively. Our results are in line with previous findings. In addition, the profiles of co-expression and regulator networks of PBK were analyzed. PPI network based on GeneMANIA showed that PBK significantly interacted with LRRC47, ARAF, LGALS9B, TTK, DLG1, and other essential genes. Biological function of these genes may include cell cycle G2/M phase transition, cell cycle checkpoint, regulation of nuclear division, mitotic cell cycle checkpoint, negative regulation of mitotic cell cycle, mitotic nuclear division and G2/M transition of mitotic cell cycle. We also obtained the co-expression genes in TCGA-LIHC that are closely related to PBK from Linkedomics. GSEA revealed the function of PBK and its related signal transduction pathway, such as cell cycle, DNA replication, amino acid metabolism and degradation, which might help explain the underlying molecular mechanisms of PBK in HCC. In the past few years, there have been few research reports that have the association between PBK with HCC. Functional analysis of PBK, derived in most cases from emerging high-throughput genomic and bioinformatics scanning approaches, is still unclear. The researchers identify PBK, a downstream effector of FoxM1, as an oncogene in HCC via the activation of β-Catenin signaling pathway in vitro and vivo [35]. Another study found PBK depletion suppressed migration and invasion of HCC cells and markedly inhibited the lung metastasis of HCC cells in orthotopic mouse model. Mechanistically, PBK enhanced uPAR expression by enhancing the binding of ETV4 to uPAR promoter to activate its transcription [4]. Cao et al. reported for the first time that overexpression of PBK relieved the apoptosis induced by Oxaliplatin and promoted the migration and invasion of Oxaliplatin-sensitive HCC cells [36].

Many studies about the possible role of PBK in tumor immune cell infiltration have emerged in recent years. In Esophageal squamous cell carcinoma, immune infiltration analysis showed that infiltration of dendritic cells was significantly negatively correlated with PBK expression levels [37]. Zhou et al. investigated the immune microenvironment in Ewing’s sarcoma patients to identify immune-related gene signatures. PBK, one of 10 hub immune-related genes signature, was determined to exhibit independent prognostic significance for Ewing’s sarcoma [38]. In HCC, the expression of PBK was significantly positively correlated with immune infiltration cells, including regulatory T cells (Treg), T follicular helper (TFH) cells, macrophages M0, but negatively correlated with immune infiltration cells including monocytes [39]. In our study, the results from the TIMER database shown that a significant positive correlation between PBK and six main infiltrating immune cells (CD4+T cells, CD8+T cells, B cells, macrophages, neutrophils, and dendritic cells). Multivariate Cox regression analysis showed that macrophages and neutrophils were also significant independent risk factors. Whether PBK affects the prognosis of patients is closely related to macrophages and neutrophils remains to be studied. Unfortunately, there is no relevant report at present. The same as our findings that PBK was associated with tumor immune cell infiltration in most cases and was especially positively correlated in KIRC, LGG, and LIHC [40]. Moreover, to our knowledge, it’s the first time to reveal that the expression of PBK is closely related to chemokines and receptors. These findings are significant for further research about the detailed mechanisms of PBK in HCC. Multiple studies have shown that CCL20 is expressed in tumor cells and tumor-associated macrophages, which can activate CCR6 on cancer cells in an autocrine manner, causing their migration and epithelial-to-mesenchymal transitio n[41]. Interestingly, PBK expression was positively closely related with CCR6 and its ligand-CCL20 in HCC from our results, which suggest that PBK may be involved in the CCL20→CCR6 axis, but still require further experimental validation. Similarly, our analysis showed that CCL19 and CCL21 combined with their receptor CCR7 were all negatively correlated with the expression of PBK. Meanwhile, researchers found that the increased expression of CCL19 and CCL21 in tumors can result in infiltration of TILs and an improved prognosis for many tumor patients. This is consistent with our results. Highly expressed PBK may be negatively correlated with the CCL19/CCL21→CCR7 axis and cause poor prognosis of liver cance r[41].

In conclusion, multiple studies have identified PBK as a crucial gene for HCC. The difference between our study and previous studies was that we reported the relationship between PBK expression and clinicopathological characteristics. We also identified the diagnostic value of PBK and as an independent prognostic factor in HCC. In addition, another advantage in our study was the first time to evaluate the correlation between PBK expression in pan-caners and tumor-infiltrating lymphocytes (TILs), chemokines, and receptors. However, there were few limitations in the present study. Experimental verification is also needed to elucidate the molecular mechanisms in vivo and in vitro. Thus, results in our research may provide useful information for prospective research on HCC immunotherapy and targeted therapy.

## Supplementary Information

**Additional file 1: Fig. S1** The expression of PBK in pan-cancers and HCC. A: PBK mRNA levels in 20 types of human cancers analyzed by Oncomine. Red means increased expression and blue means decreased expression. The numbers indicated the amounts of dataset satisfying the threshold in the colored cell. B: PBK mRNA levels analyzed by TIMER. (***P<0.001, **P<0.01, *P<0.05). C: The expression of PBK in HCC tissues from HCCDB. D: PBK expression profile in HCC based on GEPIA database. (*P<0.05) E-F: Protein expression and distribution of PBK from HPA.

**Additional file 2: Fig. S2**A: Relations between receptors and expression of PBK in pan-cancers (red is positive correlated and blue is negative correlated). B: PBK expression was positively closely related with CCR6 and CCR10, and was negatively correlated with CCR7 and CXCR1.

**Additional file 3: Fig. S3** A: Relations between chemokines and expression of PBK in pan-cancers (red is positive correlated and blue is negative correlated). B: PBK expression was positively or negatively closely related with chemokines (rho>0 indicate positively, rho<0 indicate negatively).

**Additional file 4: Table S1**. Multivariate Cox analysis of immune cells infiltration.

## Data Availability

The data used to support the findings of this study are included in the article.

## Abbreviations

HCC: Hepatocellular carcinoma
PBK: PDZ-binding kinase
TCGA: The Cancer Genome Atlas
GEO: Gene Expression Omnibus
GEPIA: The Gene Expression Profiling Interactive Analysis
TIMER: Tumor Immune Estimation Resource
TISIDB: Tumor-Immune System Interaction Database
HPA: The human protein atlas database
ROC: Receiver operating characteristic
AUC: Area under the curve
OS: Overall survival
RFS: Recurrence-free survival
PFS: Progression-free survival
DSS: Disease-specific survival
GO: Gene Ontology
KEGG: Kyoto Encyclopedia of Genes and Genomes
BP: Biological processes
CC: Cellular components
MF: Molecular functions
GSEA: Gene Set Enrichment Analysis
TIICs: Tumor-infiltrating immune cells
TILs: Tumor-infiltrating lymphocytes
