# Supplementary material for: High expression of PDZ-binding kinase is correlated with poor prognosis and immune infiltrates in hepatocellular carcinoma
Source: World J Surg Oncol. 2022 Jan 22;20:22. doi: 10.1186/s12957-021-02479-w (PMC8783494; doi:10.1186/s12957-021-02479-w)
Supplement: Supplementary file 1 — Additional file 1: Fig. S1 The expression of PBK in pan-cancers and HCC. A: PBK mRNA levels in 20 types of human cancers analyzed by Oncomine. Red means increased expression and blue means decreased expression. The numbers indicated the amounts of dataset satisfying the threshold in the colored cell. B: PBK mRNA levels analyzed by TIMER. (***P<0.001, **P<0.01, *P<0.05). C: The expression of PBK in HCC tissues from HCCDB. D: PBK expression profile in HCC based on GEPIA database. (*P<0.05) E-F: Protein expression and distribution of PBK from HPA. [file 12957_2021_2479_MOESM1_ESM.docx]

| A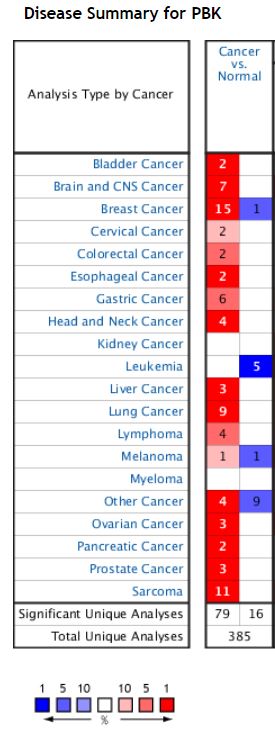 | | B  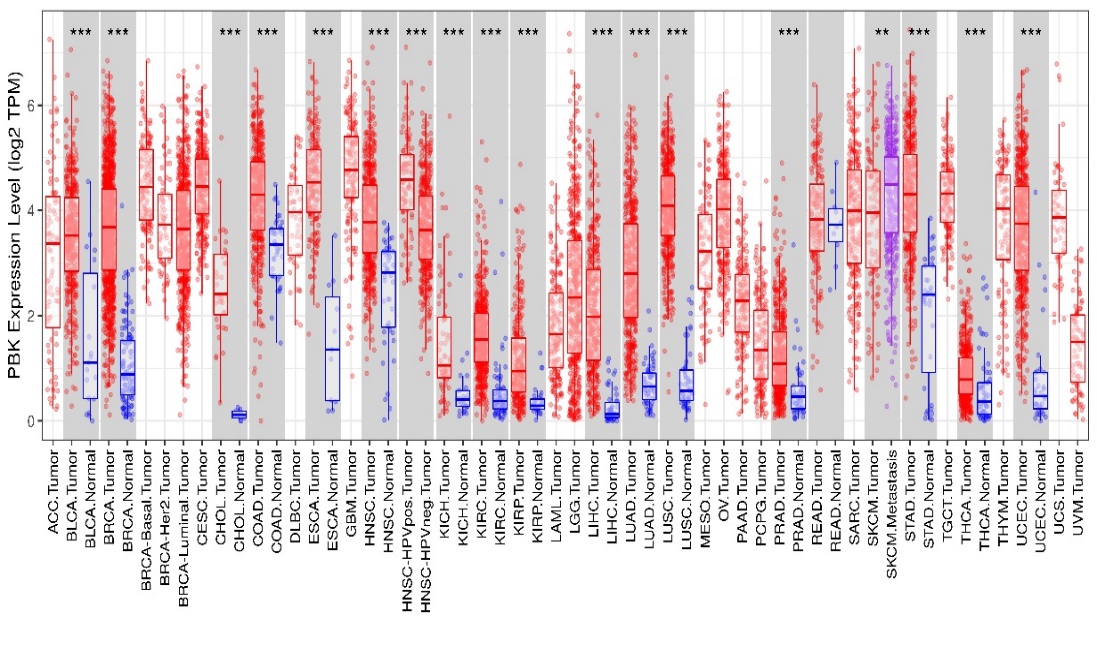 | | |
| --- | --- | --- | --- | --- |
| C | 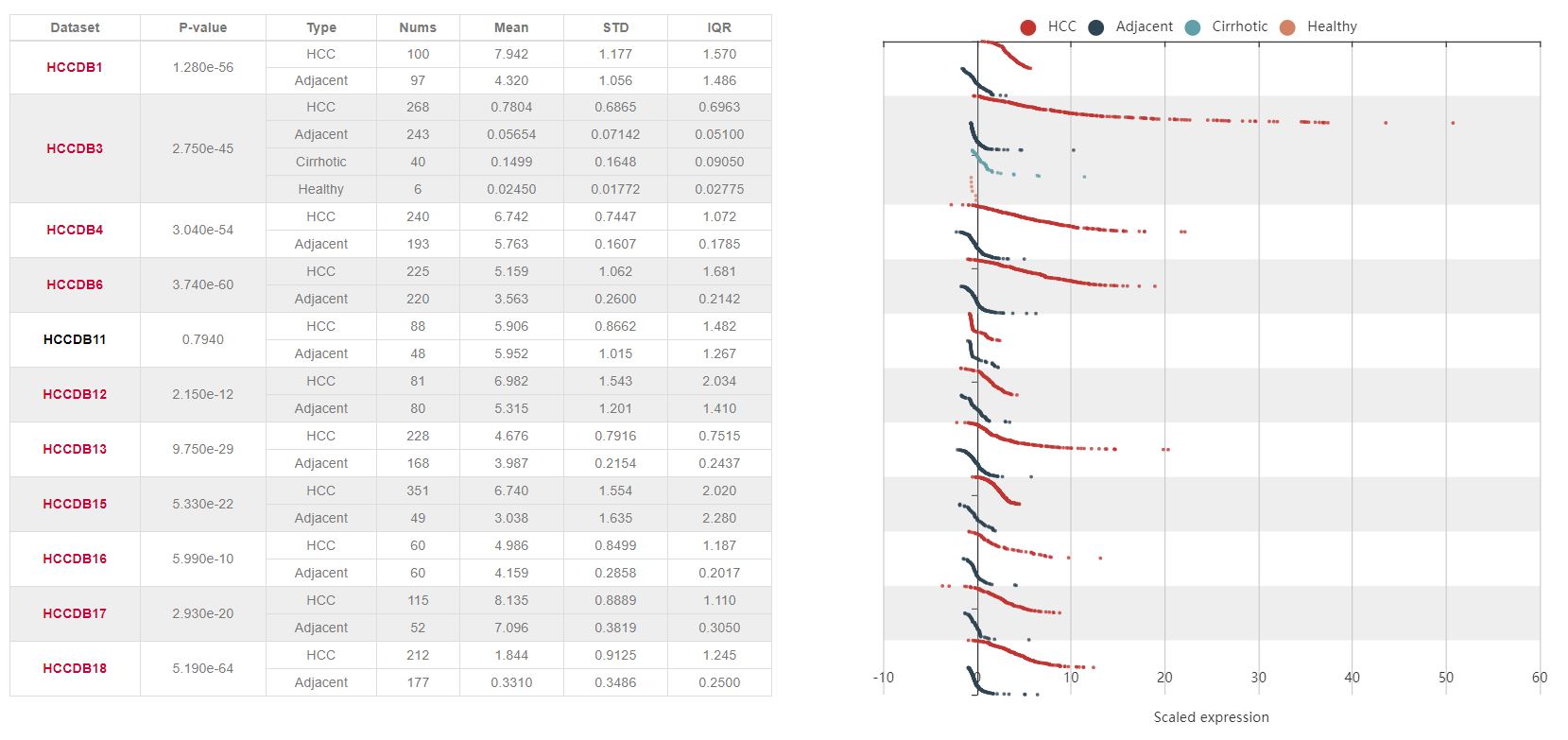 | | | |
| D  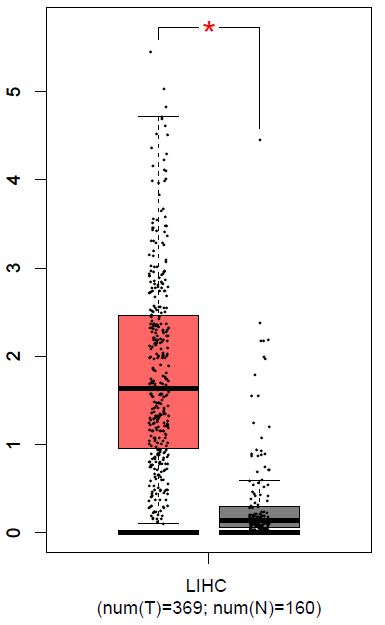 | | | E  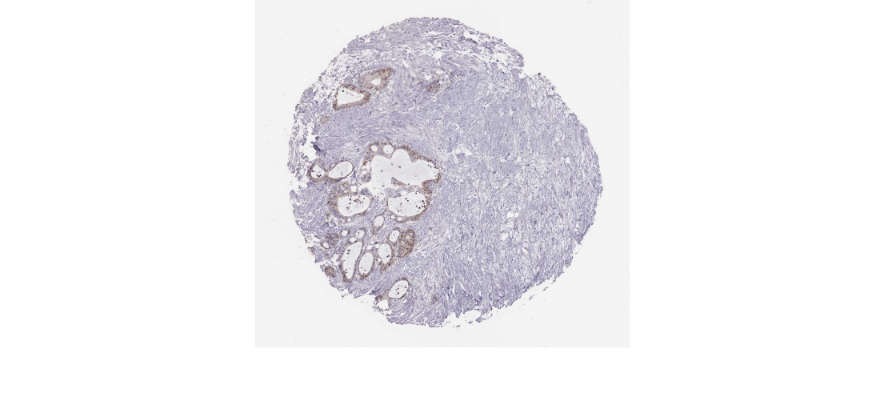  HCC  Staining: Medium  Intensity: Moderate  Quantity: 75%-25%  Location: Cytoplasmic/membranous, nuclear | F  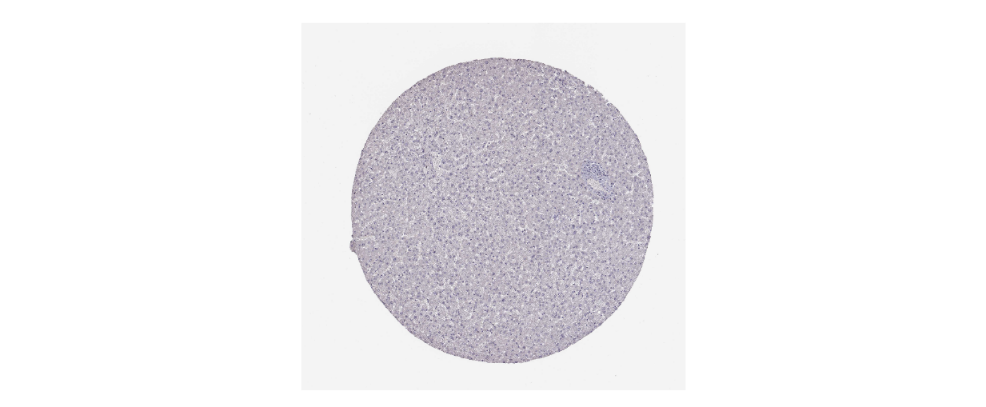  Hepatocytes  Staining: Not detected  Intensity: Negative  Quantity: None  Location: None |
| Fig.S1 The expression of PBK in pan-cancers and HCC. A: PBK mRNA levels in 20 types of human cancers analyzed by Oncomine. Red means increased expression and blue means decreased expression. The numbers indicated the amounts of dataset satisfying the threshold in the colored cell. B: PBK mRNA levels analyzed by TIMER. (***P<0.001, **P<0.01, *P<0.05). C: The expression of PBK in HCC tissues from HCCDB. D: PBK expression profile in HCC based on GEPIA database. (*P<0.05) E-F: Protein expression and distribution of PBK from HPA. | | | | |
