# Supplementary material for: High expression of PDZ-binding kinase is correlated with poor prognosis and immune infiltrates in hepatocellular carcinoma
Source: World J Surg Oncol. 2022 Jan 22;20:22. doi: 10.1186/s12957-021-02479-w (PMC8783494; doi:10.1186/s12957-021-02479-w)
Supplement: Supplementary file 2 — Additional file 2: Fig. S2A: Relations between receptors and expression of PBK in pan-cancers (red is positive correlated and blue is negative correlated). B: PBK expression was positively closely related with CCR6 and CCR10, and was negatively correlated with CCR7 and CXCR1. [file 12957_2021_2479_MOESM2_ESM.docx]

| A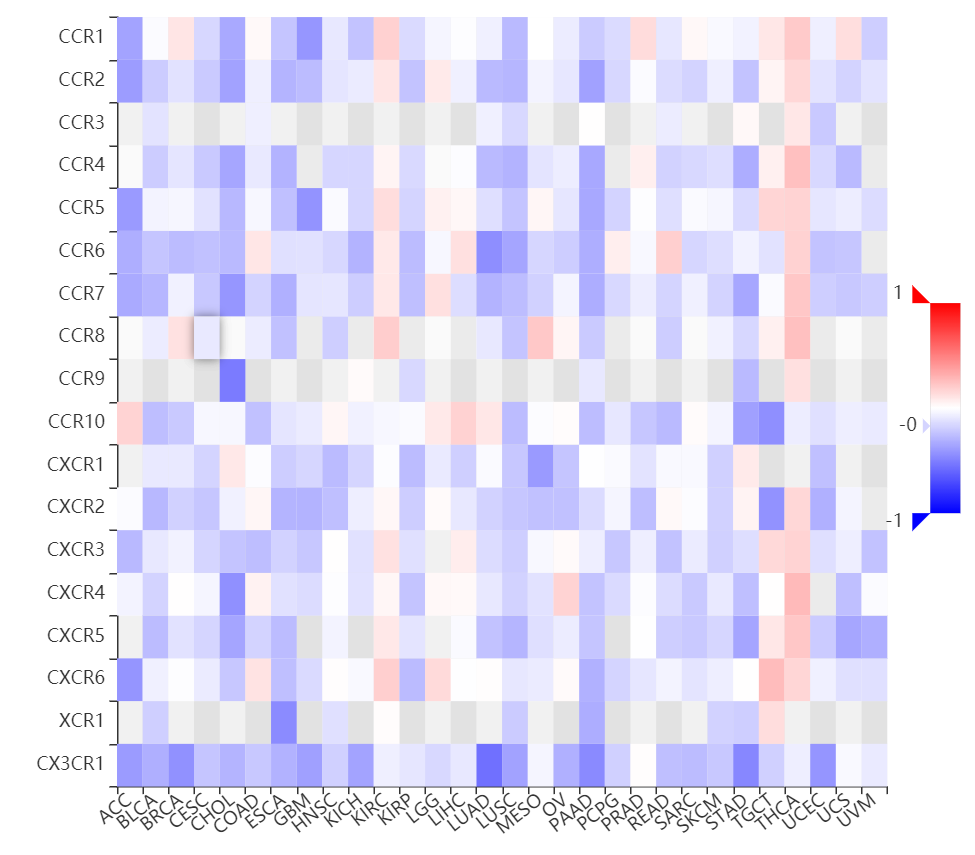 | B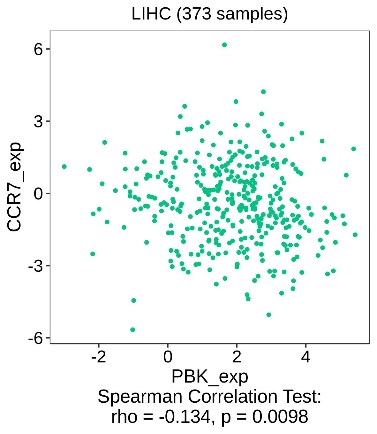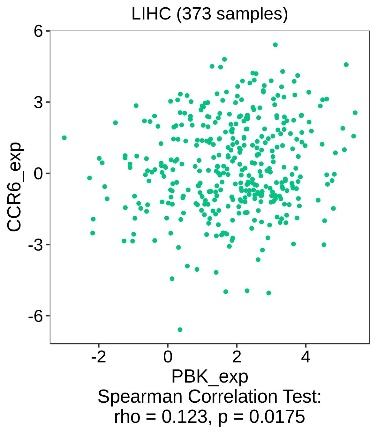 | 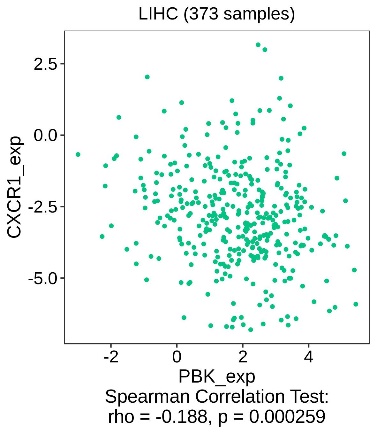  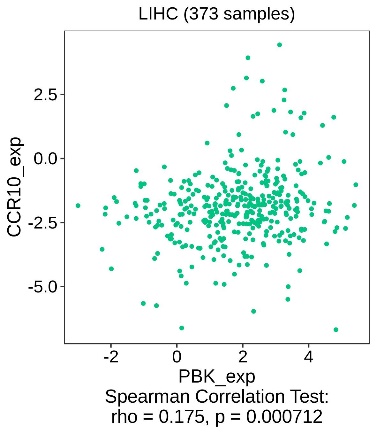 |
| --- | --- | --- |
| Fig.S2A: Relations between receptors and expression of PBK in pan-cancers (red is positive correlated and blue is negative correlated). B: PBK expression was positively closely related with CCR6 and CCR10, and was negatively correlated with CCR7 and CXCR1. | | |
