# Supplementary material for: High expression of PDZ-binding kinase is correlated with poor prognosis and immune infiltrates in hepatocellular carcinoma
Source: World J Surg Oncol. 2022 Jan 22;20:22. doi: 10.1186/s12957-021-02479-w (PMC8783494; doi:10.1186/s12957-021-02479-w)
Supplement: Supplementary file 3 — Additional file 3: Fig. S3 A: Relations between chemokines and expression of PBK in pan-cancers (red is positive correlated and blue is negative correlated). B: PBK expression was positively or negatively closely related with chemokines (rho>0 indicate positively, rho<0 indicate negatively). [file 12957_2021_2479_MOESM3_ESM.docx]

| A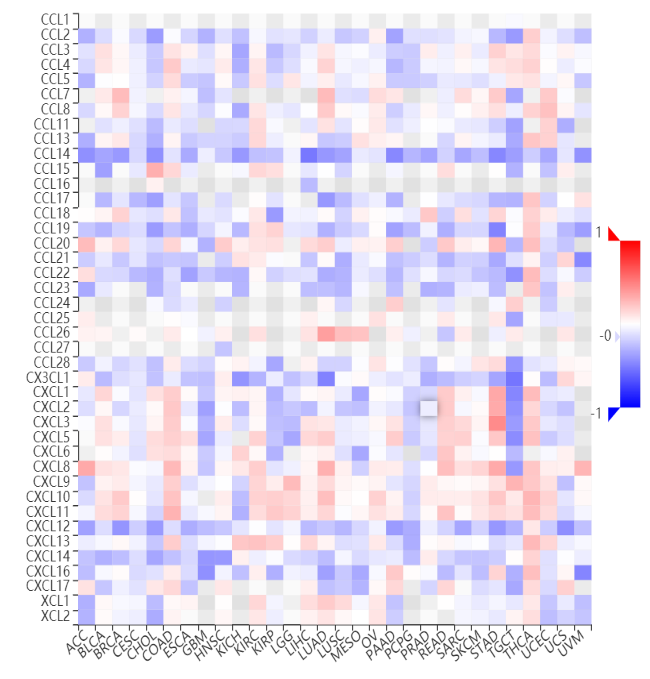 | B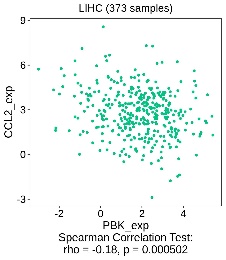 | 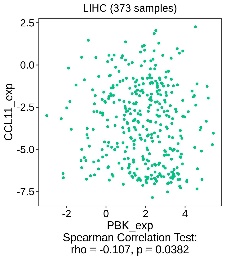 | 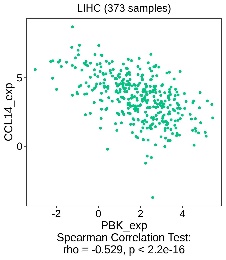 | 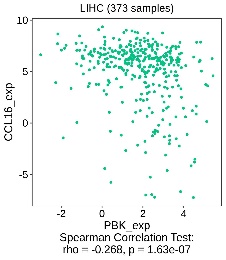 | 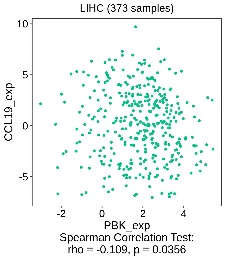 | 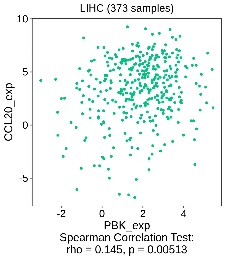 |
| --- | --- | --- | --- | --- | --- | --- |
|  | 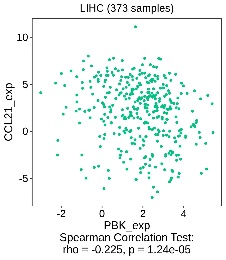 | 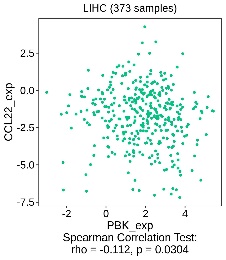 | 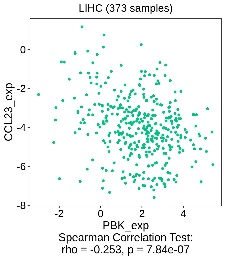 | 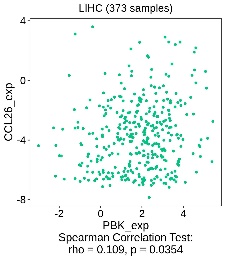 | 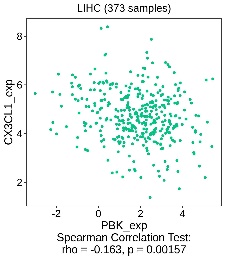 | 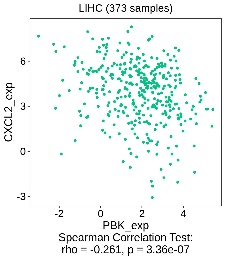 |
|  | 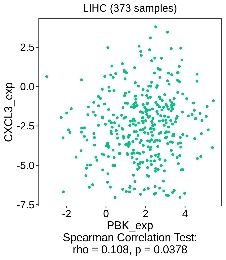 | 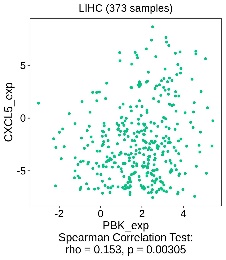 | 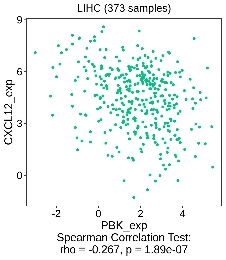 | 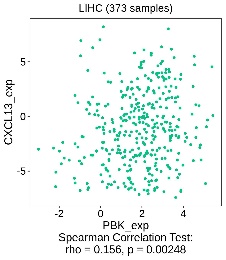 | 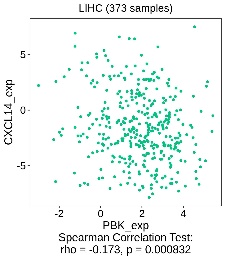 | 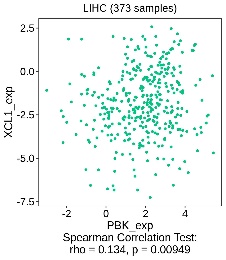 |
| Fig.S3 A: Relations between chemokines and expression of PBK in pan-cancers (red is positive correlated and blue is negative correlated). B: PBK expression was positively or negatively closely related with chemokines (rho>0 indicate positively, rho<0 indicate negatively). | | | | | | |
