# Supplementary material for: High expression of PDZ-binding kinase is correlated with poor prognosis and immune infiltrates in hepatocellular carcinoma
Source: World J Surg Oncol. 2022 Jan 22;20:22. doi: 10.1186/s12957-021-02479-w (PMC8783494; doi:10.1186/s12957-021-02479-w)
Supplement: Supplementary file 4 — Additional file 4: Table S1. Multivariate Cox analysis of immune cells infiltration. [file 12957_2021_2479_MOESM4_ESM.docx]

| Table S1: Multivariate Cox analysis of immune cells infiltration | | | | |
| --- | --- | --- | --- | --- |
| Cell type | Coef | HR | 95% CI | *P* value |
| T cell CD4+ | -0.667 | 0.513 | (0.086-3.071) | 0.465 |
| T cell CD8+ | -0.036 | 0.965 | (0.426-2.187) | 0.931 |
| B cell | -0.893 | 0.41 | (0.055-3.066) | 0.385 |
| Macrophage | 2.324 | 10.217 | (2.985-34.968) | 0.000 |
| Neutrophil | 1.56 | 4.757 | (1.303-17.361) | 0.018 |
| Dendritic cell | 0.557 | 1.745 | (0.96-3.171) | 0.068 |
| Coef: regression coefficient; HR: hazard ratio; CI: confidence interval; **P*<0.05, ****P*<0.001 | | | | |
